# Crocodiles in the Sahara Desert: An Update of Distribution, Habitats and Population Status for Conservation Planning in Mauritania

**DOI:** 10.1371/journal.pone.0014734

**Published:** 2011-02-25

**Authors:** José C. Brito, Fernando Martínez-Freiría, Pablo Sierra, Neftalí Sillero, Pedro Tarroso

**Affiliations:** 1 CIBIO - Centro de Investigação em Biodiversidade e Recursos Genéticos da Universidade do Porto, Instituto de Ciências Agrárias de Vairão, Vairão, Portugal; 2 A Cancela, Chaín 77, Pontevedra, Spain; 3 Centro de Investigação em Ciências Geo-Espaciais (CICGE) da Universidade do Porto, Porto, Portugal; University of Western Ontario, Canada

## Abstract

**Background:**

Relict populations of *Crocodylus niloticus* persist in Chad, Egypt and Mauritania. Although crocodiles were widespread throughout the Sahara until the early 20^th^ century, increased aridity combined with human persecution led to local extinction. Knowledge on distribution, occupied habitats, population size and prey availability is scarce in most populations. This study evaluates the status of Saharan crocodiles and provides new data for Mauritania to assist conservation planning.

**Methodology/Principal Findings:**

A series of surveys in Mauritania detected crocodile presence in 78 localities dispersed across 10 river basins and most tended to be isolated within river basins. Permanent *gueltas* and seasonal *tâmoûrts* were the most common occupied habitats. Crocodile encounters ranged from one to more than 20 individuals, but in most localities less than five crocodiles were observed. Larger numbers were observed after the rainy season and during night sampling. Crocodiles were found dead in between water points along dry river-beds suggesting the occurrence of dispersal.

**Conclusion/Significance:**

Research priorities in Chad and Egypt should focus on quantifying population size and pressures exerted on habitats. The present study increased in by 35% the number of known crocodile localities in Mauritania. *Gueltas* are crucial for the persistence of mountain populations. Oscillations in water availability throughout the year and the small dimensions of *gueltas* affect biological traits, including activity and body size. Studies are needed to understand adaptation traits of desert populations. Molecular analyses are needed to quantify genetic variability, population sub-structuring and effective population size, and detect the occurrence of gene flow. Monitoring is needed to detect demographical and genetical trends in completely isolated populations. Crocodiles are apparently vulnerable during dispersal events. Awareness campaigns focusing on the vulnerability and relict value of crocodiles should be implemented. Classification of Mauritanian mountains as protected areas should be prioritised.

## Introduction

The Sahara is the largest desert in the world and it is characterised by the occurrence of vast dune fields and featureless plains subjected to low precipitation levels and high temperature ranges [1]. However, this apparently bare ecosystem has not always been like this. Since the onset of the Sahara, at about 7 M.Y.A [2], its range has largely fluctuated following closely periodical climatic oscillations. Several alternated phases of dry and humid climates have occurred allowing the expansion and contraction of the desert areas, respectively, through range shifts of the hyper-arid sand seas and featureless plains [3]. At the Last Glacial Maximum (LGM, 18,000 yr), the Sahara was much larger and warmer than today, but during the mid-Holocene (7,000 yr) it was almost absent due to the higher levels of temperature and rainfall in comparison with the present day [4], [5]. During this last humid phase, the arid plains and sand seas were replaced by lakes, grasslands and open savannas in many low altitude sites, and temperate xerophytic woods and warm mixed forests covered mountains [6]–[8].

Palaeogeological events and climatic shifts constitute driving factors of current species distribution and diversity patterns in the Sahara. The progressive coolness that followed the arid LGM allowed “humid” species of Mediterranean and sub-Saharan affinity to spread over the shrunken Sahara and the mild climatic conditions of the mid-Holocene made available suitable aquatic environments for many nowadays-extinct fauna, such elephants, giraffes or hippopotamus [9], [10]. But after the Holocene, a new period of increased aridity began, that gradually dried the savannah-like ecosystems, and culminated with the revitalization of the Sahara. The disappearance of most aquatic habitats and productive savannas induced local extinction of almost all humidity-dependent species in the lower altitude areas and pushed populations to peripheral wetter regions [9], [10].

Remarkably, relict populations have persisted in mountains where suitable climatic conditions endured. Saharan mountains constitute refugia for species of Mediterranean affinity, such as the olive tree (*Olea laperrini*) and the false smooth snake (*Macroprotodon cucullatus*), and of sub-Saharan affinity, like the savannah toad (*Amietophrynus xeros*) and the Guinea baboon (*Papio papio*) [11]–[13]. Although surrounded by inhospitable desert areas, these species persist in mountain lagoons, dune lakes and high altitude mountain peaks. The isolation and small size of these habitats renders many populations vulnerable to extinction by stochastic events, loss of genetic diversity and demographic fluctuations [14]–[16]. Also, these biodiversity hotspots are currently under high vulnerability to climate changes (authors, unpub. data) and recent increased drought has been responsible for the local extinction of fish populations [17].

**Figure 1:**
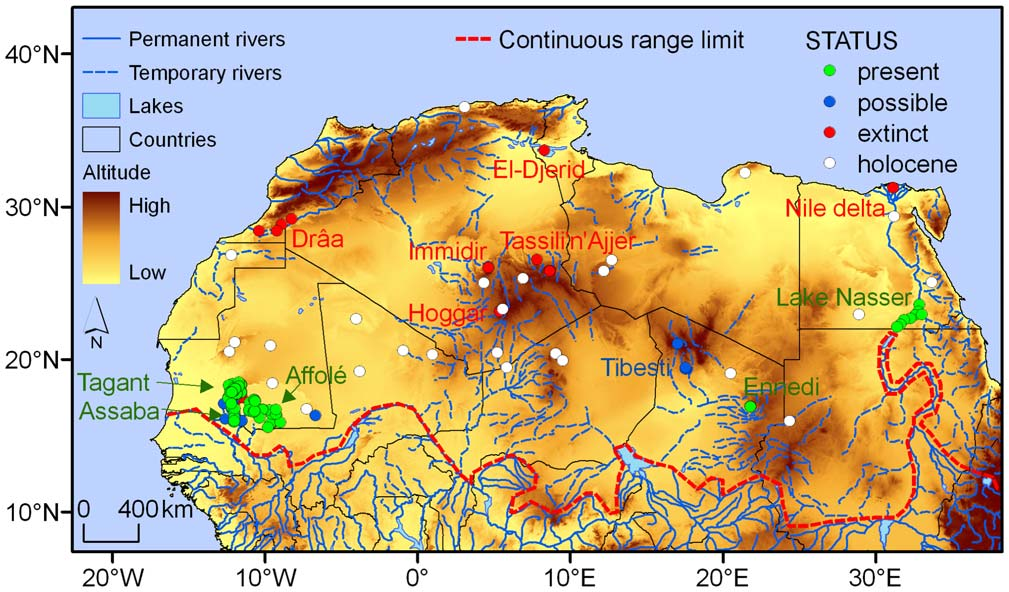
Distribution of crocodiles in North Africa. Dots represent localities where crocodiles are currently present (present), where presence is possible but needs confirmation (possible), where crocodiles were extinct in the 20^th^ century (extinct) or where crocodiles where present during the Holocene. Line represents the current northern limit of the range of continuous populations. Extinction localities georeferenced from [18], [23], [25], [55], [57]–[59]. Holocene localities georeferenced from [9], [18], [32], [42]–[54]. Possible and present localities from outside Mauritania georeferenced from [18], [22], [53], [57], [60], [64].

The Nile crocodile (*Crocodylus niloticus*) is one of the species occurring in the Sahara that experienced historical range contractions and is currently vulnerable to population isolation. Crocodiles were widespread throughout the Sahara at least from the mid-Holocene up to Roman times (Figure 1), and numerous fossil records and rock engravings depicting crocodiles are known from this period (reviewed by [18]). The increased aridity combined with human persecution has probably led to the extinction of numerous populations. By the turn of 19^th^ century, the Saharan historical exploratory missions reported their presence in the Algerian mountains of Tassili ‘n’Ajjer (reviewed by [18]), and in the 1930s, crocodiles were also found in southern Mauritanian mountains and the Ennedi massif of eastern Chad [19]–[22] (Figure 1). But soon researchers found that populations were very small and declining due to increasing human pressure [19], [23], [24]. From the beginning of the 20^th^ century until the 1960s, crocodile populations were extinct from the Chott El Djerid (Tunisia), the Hoggar and Tassili ‘n’Ajjer mountains (Algeria) and the lower Drâa river (Morocco) (reviewed by [18]). Crocodiles were present at Ihérir-Imihrou and Tedjoujelt, Tassili n'Ajjer, until the 1920s (reviewed by [18]), and an inhabitant from Ihérir village stated that he killed one specimen in 1946 [25]. Populations were also suggested to occur at I-n-Houter, Hoggar, and inquiries to locals reported their presence until the 1950s [18], [25]. Interestingly, inquiries stated the presence of crocodiles in 1984 at lake I-n-Tawinast, Immidir mountains [25]. Nevertheless, several missions held later were unable to detect the presence of crocodiles in any of these localities and the species is considered to be locally extinct [18].

In Mauritania, crocodiles were reported in the Tagant [26], but the first revision on the status of Saharan populations considered Mauritanian crocodiles to be nearly extinct, with populations probably remaining in the Assaba and Affolé mountains of southern Mauritania, and concluded that Saharan crocodile populations were virtually extinct [18]. Several scientific expeditions to Mauritania were developed from 2000 onwards [27]–[31] which reported the presence of crocodiles in three southern Mauritania massifs: Tagant, Assaba and Affolé (Figure 1). Populations are best known in the Gabbou basin, on the northern face of the Tagant mountains, where they are present in 26 isolated localities [29], [31]. But outside this river basin, knowledge on distribution is scarce. In the Affolé, crocodiles are known from four localities [27], [28], and in the Assaba, references date from before the 1970s [18], [32]–[34]. In the Tagant, crocodiles are mostly found in two major habitat types [29]–[31]: 1) rocky pools, locally known as *guelta*, generally located upstream of narrow valleys at the base of the mountains. In many cases water is only available during the rainy season (July to September), when torrential waterfalls fill up the pools. The size of gueltas varies according to the geomorphology of mountain slopes but generally are small, ranging between 0.001 ha and 1.0 ha; and 2) floodplains, locally known as *tâmoûrt*, located on the foothills of the mountains, which are larger in size and reach frequently more than 1,000 ha. Nevertheless, water is usually shallow and tâmoûrts are mostly arid during the dry season (October to June), thus crocodiles are forced to find shelter in nearby rock outcrops during this period [27], [30]. Despite the recognized importance of gueltas and tâmoûrts for the persistence of populations in the Tagant, little is known about occupied habitats in the Affolé and Assaba where crocodiles have been suggested to use distinct habitats, such as rivers (locally known as *oued*), lakes and dams [29], [32].

Crocodile populations in Mauritania are fragmented and many are constituted by a small number of individuals [27], [29]–[31], [35]. Estimates are mostly restricted to the Tagant, where one to three individuals where mentioned in small sized gueltas [31] and one to eight individuals in eight gueltas of the Krâa Naga river [29]. Larger numbers, 30 to 40 individuals, were mentioned to be present in tâmoûrts bordering Mali and Senegal [30]. Crocodiles have been reported to prey mostly upon fishes, birds, locust, frogs, and young domestic goats and sheep, and the Nile monitor (*Varanus niloticus*) has been suggested to be a predator of crocodile eggs and a prey for adult crocodiles, indicating a possible predator-prey relationship [27], [30], [36]. During the rainy season, water connections are established between many gueltas and tâmoûrts which might allow dispersal between populations through temporarily suitable corridors. In fact, movements of crocodiles during rainy season were recently suggested to occur [30], [31], but evidence remain flimsy and it is unknown if actual gene flow occurs between populations. Most likely, loss of genetic diversity and inbreeding depression due to reproductive isolation are expected to threaten these populations [37].

This study aims to evaluate the status of crocodiles in the Sahara and for Mauritania, in particular, it is aimed to update the distribution, characterise occupied habitats, provide local counts of crocodiles, and assess possible dispersal events. The results of this study are intended to increase the knowledge about distribution and occupied habitats by crocodiles in the Sahara, and to assist conservation planning in Mauritania, particularly to provide additional data for the recent listing of the “Gabbou basin” in the Ramsar Convention [38].

## Methods

Ethics statement: Fieldwork in Mauritania developed with permission from the Ministère Délégué auprès du Premier Ministre, Chargé de l’Environment. Parc National du Banc d’Arguin, Nouakchott (Permit: 460/MDE/PNBA). There are no animal husbandry, experimentation and care/welfare concerns.

### Study area in Mauritania

The study area encompasses the mountains of Tagant, Assaba and Affolé, in southern Mauritania (Figure S1). Altitude ranges from 9 m on the Senegal river valley up to 625 m in the Tagant. There is a cool, dry season from November to February and a hot, dry season from March to June [39]. Variation in annual average temperature is relatively small and tends to follow the altitudinal gradient [40]. Rain falls in a single wet season from July to October, with most precipitation in August and September [39]. There is a marked north-south gradient in annual precipitation, from 98 mm in the northern desert areas to 884 mm in the extreme southern region of the study area [40].

Most of the study area is covered by open and sparse grasslands (49.3%; [41]) with vegetation dominated by *Acacia ehrenbergiana*, *Acacia tortilis*, *Balanites aegyptiaca* and associated species [31]. Stony and sandy desert with dunes (24.7%), present only in the northern region, and croplands (17.3%), present only in the southern region, complete the most representative land cover types of the study area [41].

### Fieldwork in Mauritania

Two field missions were developed in 2008 (10 October to 18 November) and 2009 (31 March to 5 May), following previous short-timed visits in 2003 (21 to 25 November) and 2007 (9 to 16 December), totalling 109 days of fieldwork. A total of 102 localities (water points) were visited, of which 19 were visited in two or more occasions (Figure S1). Each visited locality was sampled for the presence of crocodiles by four persons (2008 and 2009 only) using a combination of distinct methodologies: 1) visual inspection of water from elevated points using binoculars and a telescope; 2) search of crocodile signs in shorelines, including faeces, footprints, tracks or burrows excavated in compact-sandy terrain (Figure S2); 3) inspection of rock crevices for hidden crocodiles; 4) night sampling of water and margins with lamps in 26 localities; and 5) inquiries to locals about the presence of crocodiles and location of dead crocodiles. The number of crocodiles present at each locality was quantified, distinguishing between day and night periods. Sampling of tâmoûrts was limited by their frequent large dimensions and most likely the number of crocodiles observed represents a small fraction of the population.

Crocodiles found dead were measured, photographed and the probable cause of death was estimated. In some cases locals informed us that the crocodile had been deliberately killed and clear beating marks were usually found in the head. Inquires were also used to complement information about annual water availability, permanent or seasonal, and the month when locality dries. To identify dispersal events, vehicle-based surveys were conducted whenever possible along the river-beds between localities with known presence of crocodiles. Riverbeds were in many occasions the main overland route between localities, which facilitated sampling and inquiries to locals. Occurrence of dispersal was considered when dead crocodiles were found along the dry river-beds in between water localities. The coordinates of localities with crocodiles and crocodiles found dead outside water points were gathered from a Global Positioning System (GPS).

### Analytical methods

Localities with presence of crocodiles were collected from bibliographic references, including for the Saharan Holocene [9], [18], [32], [42]–[54], for the Sahara excluding Mauritania [18], [22], [23], [25], [52], [55]–[60], and for Mauritania only [9], [18], [20], [26]–[29], [31]–[33], [35], [39], [43], [44], [61], [62]. These included localities with geographic coordinates or with clear geographic designations from which it was possible to gather coordinates from topographical maps (1∶200,000 from Institut Géographique National, IGN). Localities were displayed in the Geographical Information System (GIS) ArcGIS 9.3 [63] on the WGS84 datum. Locality names used in this study follow the toponomies established in the IGN maps.

Status of populations in Mauritania was ranked in four categories: 1) present, when crocodiles were observed during field missions, when faeces or footprints were found, or when recent bibliographic references (after year 2000) reported presence but field missions did not sampled these localities; 2) possible, when locals reported presence of crocodiles in apparently suitable habitats but individuals or their signs were not observed; 3) not confirmed, when localities referenced recently in bibliography (after year 2000) with presence were sampled during field missions but individuals or their signs were not observed; 4) extinct, when inquiries to locals and bibliographic references reported the extinction of crocodiles and field missions also did not found evidences for their presence.

## Results

Populations of the Nile crocodile in the Sahara are currently known from three countries, Chad, Egypt and Mauritania (Figure 1). An appreciation on the status of populations and conservation issues affecting habitats are given below. Summary data on Saharan localities (excluding extant populations in Mauritania) are given in Table S1.

### Chad

Populations of crocodiles in Chad are best known from Guelta Archei in the eastern Ennedi mountains, where several individuals have been reported since the 1930s (Figure 1) [18], [22], [57], [60], [64]. There are no quantifications of population size, but crocodiles were stated to occur in large numbers in the 1950s (reviewed by [22]). Two specimens were photographed in the 1990s [22] and another one in 2007 [60]. Crocodiles have been also suggested to occur at gueltas Oudougeï and Tottous (reviewed by [18]), but presence lack confirmation due to the regional conflicts that hamper the access to the Tibesti mountains.

### Egypt

Crocodiles were abundant in the Nile delta up to the 1800s, but by the beginning of the 20^th^ century they became largely restricted to the Nile south of Aswan and probably went extinct during the 1960s (Figure 1) [52]. After the completion of the High Dam and the filling of Lake Nasser in the late 1960s, suitable habitats were created and crocodiles returned [52]. Colonisation probably occurred with dispersal individuals arriving from populations in The Sudan. Population size is estimated to be considerably less than 5,000 breeding adults [52], and recent surveys counted an average of 71.5 crocodiles per 100 km of shoreline sampled [65]. Conflicts with growing human activities in the region are increasing, particularly with fishers, and crocodiles are locally hunted for pet trade and skins [52], [65].

### Mauritania

**Figure 2:**
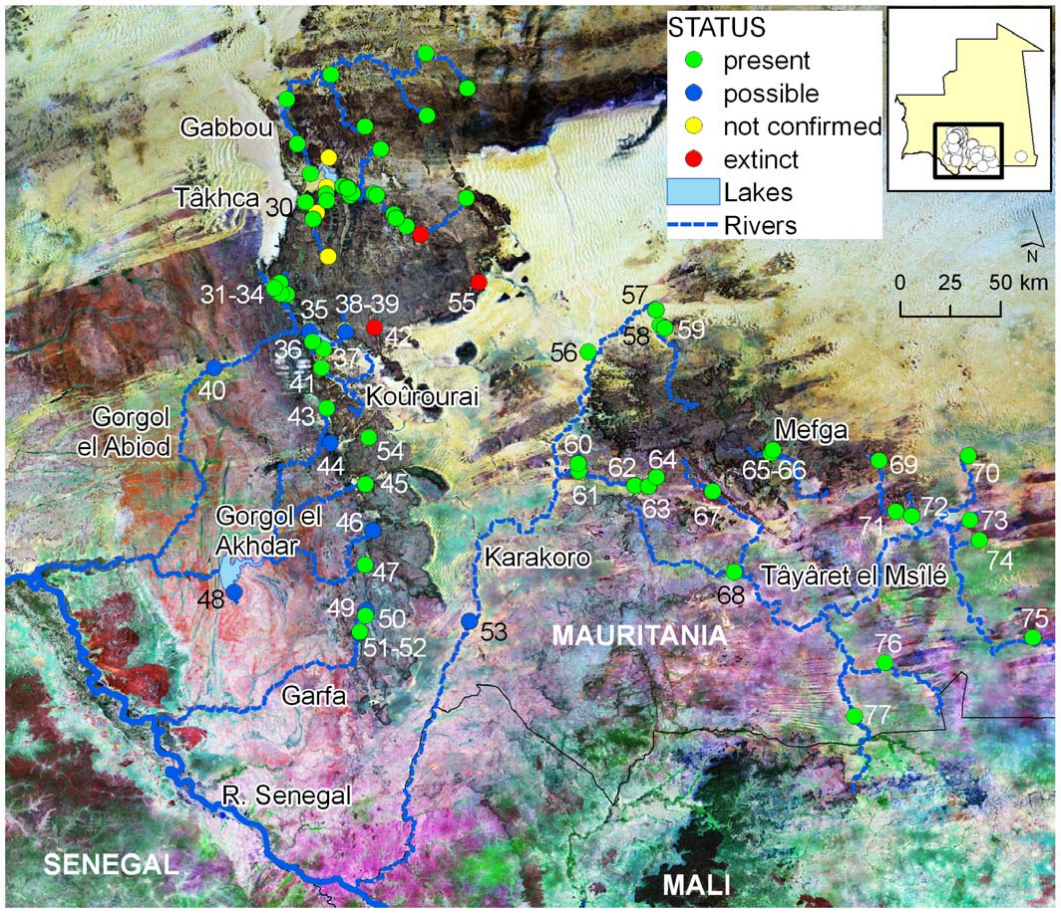
Distribution of crocodile localities along major river basins of southern Mauritania. Dots represent localities where crocodiles are currently present (present), where presence is possible but needs confirmation (possible), where crocodiles were reported to be present but this study did not confirmed their presence (not confirmed) or where crocodiles went extinct (extinct). Easternmost known locality in Mauritania (locality 78) is only represented in small inset. Numbers refer to localities described in Table S2. Localities for Gabbou basin are shown in detail in Figure 3. Background is a composite Landsat image depicting land-cover.

**Figure 3:**
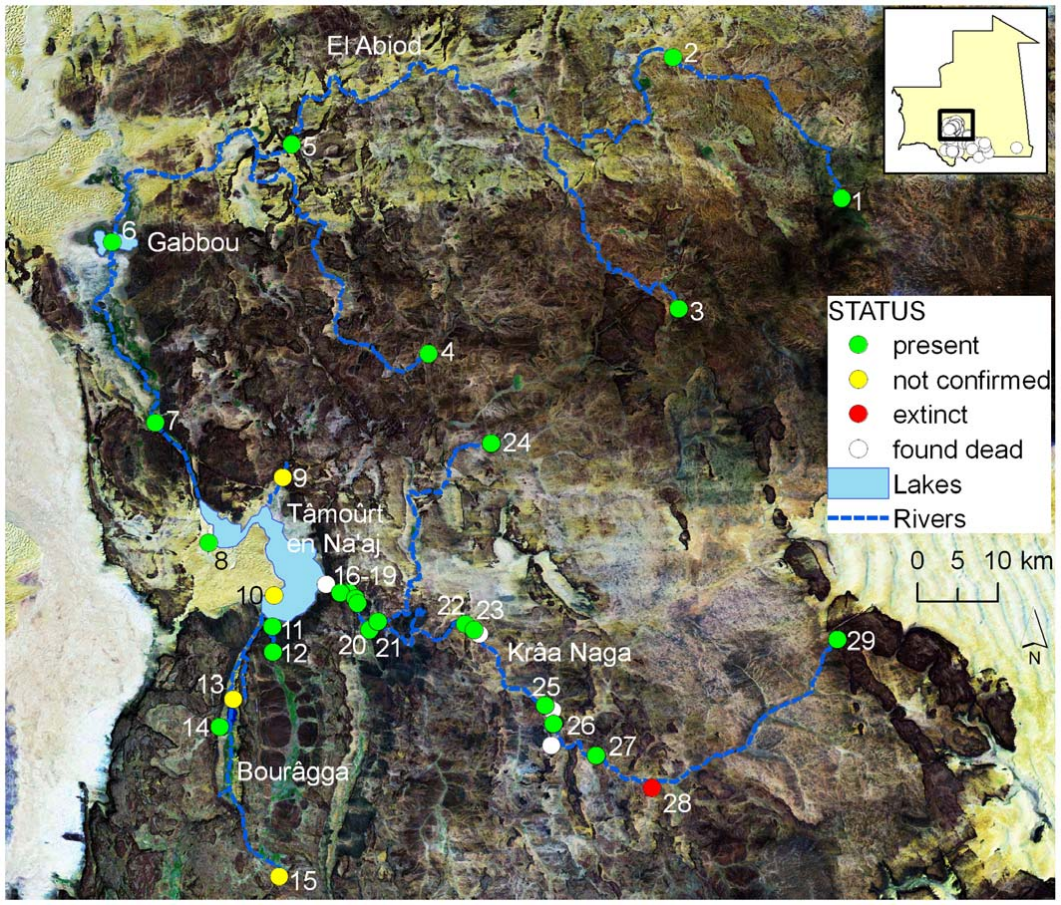
Distribution of crocodile localities along the Gabbou river basin on Tagant mountains. Dots represent localities where crocodiles are currently present (present), where crocodiles were reported to be present but this study did not confirmed their presence (not confirmed) or where crocodiles were found dead (found dead). Numbers refer to localities described in Table S2. Names represent major seasonal water lines flowing to the Gabbou lake. Background is a composite Landsat image depicting land-cover.

Field missions and bibliographic references identified 78 crocodile localities in Mauritania (Figures 2 and 3; Table S2). Of these, crocodiles were found to be present and possibly present in 60 and 11 localities, respectively, whereas their presence was not confirmed in four previously known localities, and confirmed as extinct from three localities. Presence was for the first time reported in 27 localities, meaning that this study increased in 35% the number of known localities with crocodiles in Mauritania.

**Figure 4:**
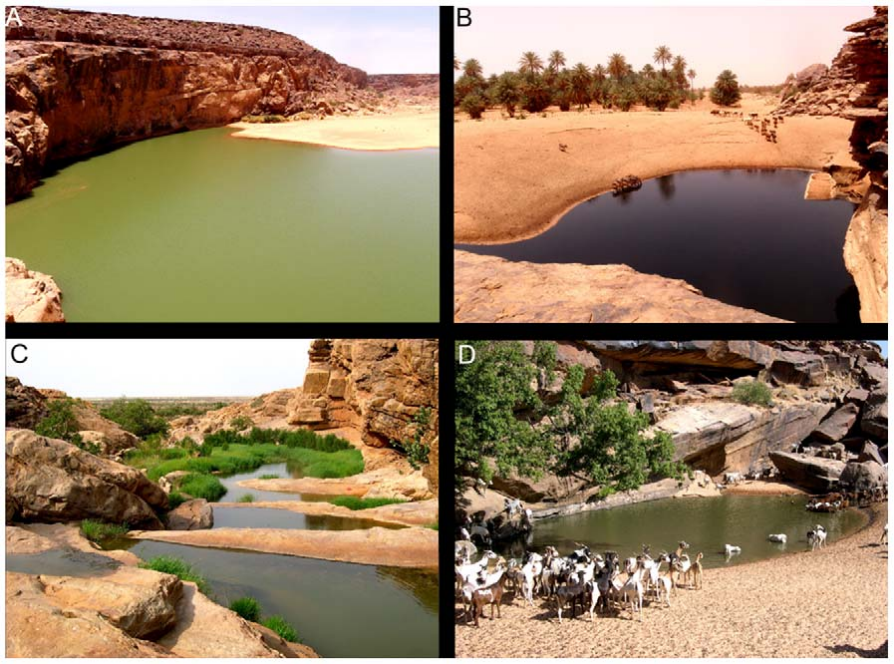
Examples of gueltas with presence of Nile crocodiles in Mauritania. A. Tartêga; B. El Khedia; C. Garaouel; D. Oumm el Mhâr.

**Figure 5:**
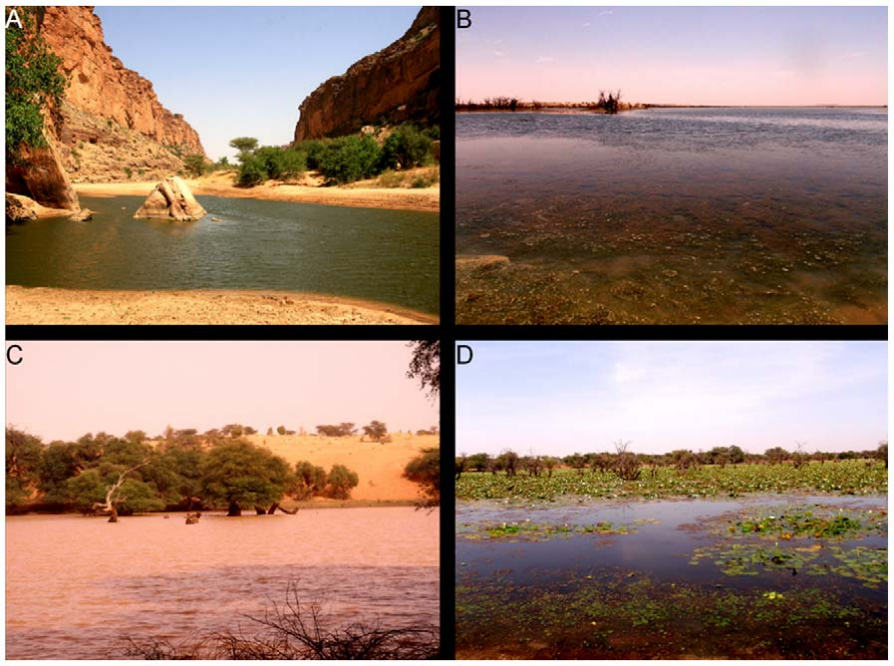
Examples of oueds, lakes and tâmoûrts with presence of Nile crocodiles in Mauritania. A. oued Foum Goussas; B. lake Boû blei'îne; C. tâmoûrt Taghtâfet; D. tâmoûrt Bougâri.

Excluding localities where crocodiles were not confirmed or extinct, gueltas and tâmoûrts were the most common habitats where individuals were found (40.8% and 26.8%, respectively), but presence was detected also in oueds (9.9%), sources (8.5%), lakes (8.5%) and dams (5.6%) (Figures 4 and 5). Crocodiles were observed in 19 permanent gueltas (73% of gueltas surveyed) and 17 seasonal tâmoûrts (90% of tâmoûrts surveyed).

**Figure 6:**
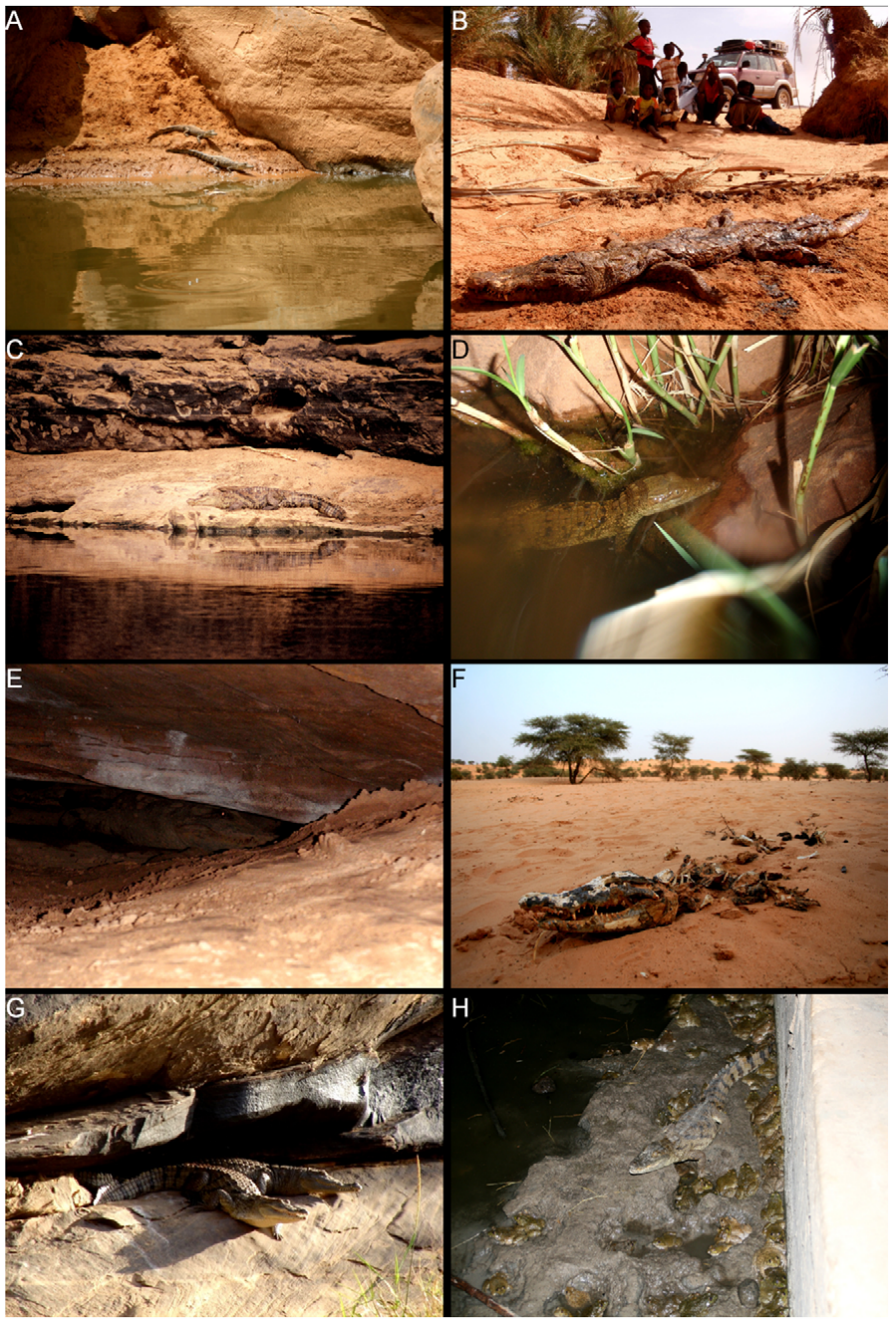
Examples of Nile crocodiles from Mauritania. A. juveniles at guelta Matmâta; B. adult killed at Dar-Salam village, near guelta Matmâta; C. the single adult at guelta El Khedia; D. sub-adult at guelta Garaouel; E. two adults hidden in a cave at 8 m depth in guelta Legleyta; F. adult killed near tâmoûrt Taghtâfet; G. basking adults at guelta Metraoucha; H. sub-adult inside a spring-fed water trough at Chegg el Mâleh surrounded by hundreds of frogs (*Hoplobatrachus occipitalis*).

The total number of crocodiles observed was 178, including yearlings to adults (Figure 6). The number of observations ranged from one to more than 20 individuals in 33 sampled localities. The smallest population was the single adult present in guelta El Khedia (Figure 6C). In most localities, less than five crocodiles were observed (N = 17) and seven of these localities corresponded to gueltas. Most localities where more than 10 crocodiles were observed had permanent water (N = 5) and all of these corresponded to gueltas.

Four localities along the Krâa Naga oued (gueltas Amzouzef, Ch'Bayer, Matmâta, and Tartêga) were sampled in the period 2000–2002 [29] and the average (±standard deviation) number of crocodiles detected was 2.8±3.5. This study sampled these localities in the period 2008–2009 and the number of individuals observed was 6.5±3.1.

A larger number of crocodiles were observed after the rainy season in comparison with the dry season. In localities with crocodiles sampled in late 2008 (N = 20), an average of 5.9 individuals were observed, whereas in localities sampled in early 2009 (N = 13), an average of 3.5 crocodiles were observed. Also, in localities sampled during daylight and at night (N = 20), more crocodiles were observed on average at night (6.1±5.3) than during daylight (2.7±4.6).

A total of 10 crocodiles were found dead, of which five had been killed by locals, two died from apparently natural reasons and three by unknown reasons (only bone remains were found after digging). Seven of these crocodiles were found in between water points along the dry river-bed and were considered to be dispersing (Figure 6B and 6F). All dispersing individuals found were sub-adults and adults with body size larger than 1.20 m. Five dispersing crocodiles were found along the Krâa Naga oued (Gabbou basin, Tagant) where gueltas are usually found at relative short distances (on average less than 4 km).

The 78 localities are dispersed across 10 river basins and most tended to be isolated within river basins (Figure 2). Summary data on localities, population status, and date of last observation are given in Table S2. Detailed data on localities, crocodile observations, population status, and conservation issues affecting habitats in Mauritania are given in Text S1.

## Discussion

Nile crocodiles occur in the Sahara desert in fragmented populations throughout several mountains. Although the mechanisms explaining the presence of crocodiles in the Sahara are well understood (e.g.[10]), in reality there is paucity of knowledge about distribution, demography, ecology, and conservation status of populations. Research priorities in Chad and Egypt should focus on studies quantifying population size and pressures exerted on habitats in the population present in guelta Archei and Lake Nasser (e.g.[65]). Field surveys are also needed in the Tibesti where the presence of crocodiles is uncertain [56]. The remoteness and isolation character of these mountains might have assured the persistence of crocodiles. Fine-scaled remote sensing techniques might be applied prior to fieldwork in order to identify suitable water localities for the occurrence of crocodiles [66]–[68].

The present study increased in by 35% the number of known crocodile localities in Mauritania. Presence was confirmed in 60 localities and another 11 were identified as of possible presence. The increase in known localities is probably related to previous lack of sampling and cryptic behaviour of crocodiles. The remote character of southern Mauritanian mountains, associated to with logistical fieldwork constraints, has prevented detailed sampling. Also, crocodiles were found spending large portions of time hidden inside caves or burrows [27], [30], [31], further hampering their detection (e.g. Figure 6E). Thus, it is likely that increased sampling will detect more populations. The southern Gorgol el Abiod, Gorgol el Akhdar, Garfa and Karakoro basins should be further surveyed as suitable areas may be present. Sampling should also be aimed to extreme south-eastern Mauritania, where besides lake Dendaré (locality 78), no other localities are known, but water availability (e.g. Mahmoûdé lake) may allow crocodile presence. Assessment of population status along the Senegal river is also needed, where accidental death in fishnets and direct harvesting may have severely reduced populations and restricted crocodiles to local suitable areas, such as the National Parks of Diawling (Mauritania) and Djoudj (Senegal) [28], [35], [69]. While local beliefs of the Moor ethnic group protect mountain-ranging crocodiles [27], [28], [30], the southern Mauritania ethnic groups hunts them for skin, organs and meat, along the Senegal river and major tributaries [28], [36]. The increasing human pressure is also the most likely responsible for the extinction of the Slender-snouted crocodile (*Crocodylus cataphractus*), which was reported along the Senegal river [36], [70], but currently is considered extinct [28].

Crocodiles were mostly found in gueltas and tâmoûrts, which is probably related to their higher abundance in comparison to other water habitats. Gueltas are apparently crucial for the persistence of crocodiles in mountains, as already emphasised for other vertebrates with isolated populations [15]–[17]. Although population size is unknown, relatively low number of individuals observed in almost all localities (on average less than five individuals were observed at each locality), but particularly at gueltas, suggests that the actual number of crocodiles present is small, which stresses the vulnerability of these habitats to threat factors. After the severe droughts of the 1970s [71], [72], there were large human movements and settlement around water localities [30]. Currently, several gueltas are overexploited by herdsmen, producing water-shortage during the dry season, faecal contamination by domestic animals, and increased activities for excavating pools or pumping water [31]. Furthermore, several small-sized gueltas were strongly disturbed by drinking cattle during daylight (Figure 4D). Although crocodiles tend to be more active at night [73], increased human activities during daylight force individuals in small-sized gueltas to remain hidden, while at night they are able to explore the surroundings of lagoons. Local studies should be conducted to quantify pressures and threat risks affecting crocodile populations.

Oscillations in water availability throughout the year and the relatively small dimensions of gueltas have dramatic consequences in the activity and ecology of populations. During the dry season, individuals are forced to aestivate [73]. In gueltas, they find shelter between the rock boulders of the rocky slopes, as observed in guelta Legleyta (Figure 6E), while in tâmoûrts, they burying themselves below the mud surface or migrate to nearby rock outcrops, as observed in tâmoûrt Bougâri [27], [28], [30]. Apparently, crocodile presence is more frequent in tâmoûrts with available rock outcrops within a 5 km perimeter [28]. Activity is concentrated in the period when water is available and more crocodiles were observed after the rainy season than during the dry season. Apparently, mountain populations of crocodiles have the feeding, growth and reproductive period restricted to just about 10 weeks per year (or even less at some localities). During the rainy season and the following weeks, prey availability may increase dramatically, as observed at Chegg el Mâleh (Figure 6H and S3): in November, there were hundreds of active amphibians (*Hoplobatrachus occipitalis*), while in December, no amphibians were detected and the tâmoûrt was dry, suggesting that crocodiles should feed only during the active period of amphibians. The relatively short feeding period and available prey of small size, mostly fishes and amphibians, probably affects growth rates and reduces maximum body size [30], [73]. All crocodiles observed were less than 3 m long, and the vast majority were less than 2 m (Figure 6G). The largest crocodiles were observed in guelta Tartêga, which has permanent water and is one of the biggest gueltas of Mauritania (Figure 4A). Although hatchlings or nests were not found, yearling crocodiles were observed at Matmâta and sub-adults were observed in several localities (Figure 6A and 6D), suggesting that reproduction occurs in isolated populations. Demographical, behavioural and thermoregulatory studies should be conducted to understand adaptation traits of these populations to extreme environmental conditions.

Dead crocodiles were found between water points along dry river-beds, suggesting the occurrence of dispersal (Figures 6B and 6F). Movement of crocodiles during the rainy season along temporary water connections have been suggested to occur in mountain populations. A displacement of a crocodile of 2 km over dunes has been reported [30] and potential extinctions in small gueltas, with 1 to 3 individuals, have been suggested that could be compensated by the arrival of animals moving along river beds [31]. Possible population connectivity could occur along the Krâa Naga and upper Karakoro basins, where there are several gueltas and tâmoûrts, respectively, located at relatively short distances (e.g. Ch'Bayer and Rh' Zembou in the former, and Taghtâfet, Jaraaziza, Tâmchekket in the latter). Also, tâmoûrt Djouk is of special relevance given that it may assure connectivity between populations located in the Tagant and Assaba mountains. Thus, it can be hypothesized that Mauritanian crocodiles form a metapopulation, where loss of genetical diversity in lagoons could be attenuated by the occasional migration of individuals with associated gene flow. Furthermore, dispersal from mountain lagoons to the Senegal river may also occur through the Gorgol and Garfa rivers but supporting evidences are needed. Use of molecular markers is necessary to quantify genetic variability, population sub-structuring and effective population size, and detect the occurrence of gene flow.

The present study identified localities completely isolated without any possibility of rescuing-effects [74]. This is the case of source Oumm Icheglâne and guelta Legleyta, which are isolated within the Assaba mountains (Figure S2 and S3). The most dramatic case occurs at guelta El Khedia (locality 29; Figure 3 and 6C), where a single adult is the remaining exemplar from a larger population (reviewed by [18]) and the nearest population is relatively distant (37 km). Isolation by distance apparently prevents dispersal between water localities. For instance, guelta Mendjoura had crocodiles until severe droughts in the 1970s induced local extinction. The closest locality with crocodiles, Boû blei'îne (Figure 5B), is at over 60 km and the connecting oued is totally covered by dunes. Thus, although the guelta currently presents reasonable water levels and prey is available, large distances and unsuitable habitats apparently hamper colonisation. Monitoring of effectively-isolated populations is needed to detect demographical and genetical trends. Introduction of specimens from nearby relatively dense populations should be considered for El Khedia.

The isolation and vulnerability of mountain populations apparently induces behavioural shifts in aggressiveness patterns of crocodiles. Individuals are extremely shy and plunge into water at the first sign of human disturbance. Interestingly, this behaviour was also reported for the extinct Algerian populations [24]. In Mauritania, inquires did not indicate crocodile attacks to humans and, as previously observed by Shine et al. [27], locals swim and wash in gueltas with crocodiles. Even so, when more than one lagoon was available, humans used preferentially the lower ones and crocodiles were more numerous in the upper ones. Although local beliefs protect crocodiles [27], [28], [30], these are apparently killed whenever found far from the gueltas, probably during dispersal events. Local public awareness campaigns focusing on the vulnerability and relict value of crocodile populations should be implemented.

Climate change scenarios for the region predict significant warming and rainfall decrease [75], [76], which are expected to increase population isolation and local extinction (authors, unpub. data). Multi-scale conservation strategies are needed to protect populations and mitigate climate change effects [77]. Classification of Mauritanian mountains as protected areas should be prioritised, as these should contribute to minimise human induced land transformation and habitat loss [78], which are also important threats to local biodiversity.

## Supporting Information

Text S1 Detailed data on localities, crocodile observations, population status, and conservation issues affecting habitats in Mauritania.
(0.07 MB DOC)

Table S1 Distribution, status and date of last observation of Nile crocodile populations in the Sahara excluding extant localities for Mauritania.
(0.10 MB DOC)

Table S2 Distribution, status and date of last observation of Nile crocodile populations in Mauritania.
(0.16 MB DOC)

Figure S1 Distribution of sampled localities in the three main mountains of southern Mauritania. Dots represent sampled localities (water points). Only the river basins (names) with crocodile populations are presented.
(1.77 MB TIF)

Figure S2 Presence signs of crocodiles in Mauritania. Tracks and footprints (A) at tâmoûrt Kour, faeces (B) at guelta Garaouel and burrows (C) at tâmoûrt Bougâri were taken as presence signs of crocodiles.
(6.55 MB TIF)

Figure S3 Photos of extremely small localities with presence of Nile crocodiles in Mauritania. A. Five to six crocodiles occur at source Aouînet Nanâga; B. two juveniles were observed at source Oumm Icheglâne; C. one adult and two sub-adults were observed inside a spring-fed water trough at Chegg el Mâleh.
(6.54 MB TIF)
