# Supplementary material for: Crocodiles in the Sahara Desert: An Update of Distribution, Habitats and Population Status for Conservation Planning in Mauritania
Source: PLoS One. 2011 Feb 25;6(2):e14734. doi: 10.1371/journal.pone.0014734 (PMC3045445; doi:10.1371/journal.pone.0014734)
Supplement: Text S1 — Detailed data on localities, crocodile observations, population status, and conservation issues affecting habitats in Mauritania. (0.07 MB DOC) [file pone.0014734.s001.doc]

**Text S1**

Detailed data on localities, crocodile observations, population status, and conservation issues affecting habitats in Mauritania.

**A) Gabbou basin**

The endorreic basin flows to the Gabbou lake and crocodiles are known from four major water courses: Oued el Abiod, Bourâgga, Krâa Naga and Tâmoûrt en Na’aj.

1. Ederoum (dam) - present

The permanent dam is located in upstream Oued el Abiod and crocodiles were reported since the 1990s [1,2]. In 1993, five crocodiles (0.9 to 1.0 m) were observed and in 2002, three to four individuals were reported according to locals [2]. The locality was visited in October 2008 but no crocodiles were observed. Nevertheless, several footprints were found and locals mentioned the presence of several adults and juveniles. The nearest locality, guelta Motoboul (locality 2), is at 28 km which suggests that populations at Ederoum are probably isolated.

2. Motoboul (guelta) - present

Crocodiles were reported in the seasonal guelta [3]. The locality was not visited during this study.

3. Taorta (guelta) - present

Crocodiles were reported in the seasonal guelta located in upper Oued el Abiod [3]. The locality was visited in April 2009 and crocodiles were not found. The guelta was dry which probably hampered activity. Locals confirmed the presence.

4. Kaimel (guelta) - present

Crocodiles were reported in the seasonal guelta (0.03 ha) [3]. The locality was visited in April 2009 and crocodiles were not observed. The guelta was dry.

5. Daal (guelta) - present

Tellería et al. [3] reported the presence of crocodiles in the seasonal guelta of oued El Abiod. In March 2009 the guelta was almost dry and no crocodiles or their signs were observed.

6. Gabbou (lake) - present

Crocodiles have been recently reported at Gabbou lake [3]. The area was visited in November 2008 and April 2009, and although no crocodiles were observed, locals reported its presence. The lake is relatively wide (2,500 ha) and covered with vegetation that hampers detection.

7. Dekheïlet el ‘Aleïb (lake) - present

Crocodiles were reported in the permanent lake (1,500 ha) [3]. The locality was not visited during this study. The lake is located almost half-way between Gabbou lake and Tâmoûrt en Na’aj (localities 6 and 8, respectively) and could be used as a corridor between these localities.

8. En Na'aj (tâmoûrt) - present

Crocodiles were reported to occur in the tâmoûrt [3]. The area was visited in November 2003 and 2008, and April 2009, and although crocodiles were not observed, locals reported their presence. The large size of the lake (200 ha) and difficult accessibility to the margins hamper the observation of individuals. Possibly, the lake may be used during the rainy season by dispersing crocodiles and does not hold permanent populations. Further studies are needed to confirm this hypothesis.

9. Fanar (guelta) - not confirmed

The seasonal guelta is located on the extreme north of Tâmoûrt en Na’aj (locality 8) and was reported to hold crocodiles [4]. No crocodiles or their signs were observed in November 2008 in the guelta or along the 2 km oued that descends the valley. Locals only reported the presence of Nile Monitor lizards (*Varanus niloticus*). Probably this guelta is only temporarily used during the rainy season. Permanent presence lacks confirmation.

10. Marshra (lake) - not confirmed

The permanent lake (1,000 ha) is located in the extreme south-west of Tâmoûrt en Na’aj (locality 8) and crocodiles were reported [3]. The locality was visited in April 2009 and no crocodiles were observed. As with the main tâmoûrt, it is possible that the lake is used during the rainy season by dispersing crocodiles. Further studies are needed to confirm this hypothesis.

11. Suklan (guelta) - present

Crocodiles were reported in the seasonal guelta [3], located south of Tâmoûrt en Na’aj (locality 8). In November 2008, no active crocodiles were observed but footprints and dry faeces were found. The guelta was almost dry which suggests that crocodiles were no longer active.

12. Tkhsutin (guelta) - present

The permanent guelta (0.075 ha) is located on the rocky slopes of southern Tâmoûrt en Na’aj (locality 8) and crocodiles have been reported to occur [3]. The locality was visited in November 2008 and sampled only during daylight. No active crocodiles were observed but faeces were found. Guelta Suklan (locality 11) is at 3 km distance.

13. Bourâgga (lake) - not confirmed

Crocodiles were reported in the permanent lake (1,000 ha) [3]. The locality was visited in November 2008 and April 2009 and crocodiles were not found. Several inquiries along the Bourâgga valley only reported crocodiles for the source of El Housseînîya (locality 14), but it is possible that occasional crocodiles dispersing from the source reach the lake.

14. El Housseînîya (source) - present

Crocodiles were reported in the permanent source (<1 ha) located in oued Bourâgga [3]. The locality was visited in November 2008 and April 2009 and, although no crocodiles were observed, it was found footprints and faeces, and locals confirmed presence. The main lagoon of the oasis is surrounded by thick date palms which provide efficient cover and hamper the observation of individuals. Villagers from El Housseînîya profit from the oasis water to develop agriculture, causing disturbance in the source during the day.

15. Dâber (guelta) - not confirmed

Crocodiles were reported in the permanent guelta (0.06 ha) located on the upper oued Bourâgga [3]. The locality was visited in April 2009 but no crocodiles or signs were observed. Locals also reported absence of crocodiles. The current presence needs confirmation.

16. Matmâta (guelta) - present

Crocodiles have been reported in the complex of gueltas Matmâta-Tartêga since the 1920s [1,2,3,5,6,7,8,16]. The species was considered locally extinct in 1996 given that locals presented the head of the alleged last crocodile of the guelta [5], but crocodiles were reported afterwards [2,3,8]. In this study, quantifications of crocodiles were made separately for the two gueltas. Locality Matmâta included the first small guelta until locality Jabara (locality 17), and Tartêga (locality 18) included only the largest lagoon at the end of the canyon. Matmâta was visited in three occasions: 1) in November 2003, one adult was observed by daylight; 2) in November 2008, five adults were observed during daylight and night sampling; and 3) in April 2009, 10 crocodiles were observed at night, including six adults and four juveniles. Juvenile crocodiles were observed in April swimming independently from parents (Figure 6A). West of Matmâta lays tâmoûrt en Na’aj (locality 8), at less than 2 km, suggesting that dispersion between the guelta and the tâmoûrt could occur. In fact, one adult (1.5 m) killed by locals was found just in front of Dar-Salam village (Figure 6B). The individual was about 2 km west of the guelta, already inside the tâmoûrt, and was trapped in a drying pool.

17. Jabara (guelta) - present

The seasonal guelta (<1 ha) is located halfway between gueltas Matmâta and Tartêga (localities 16 and 18, respectively) along the Krâa Naga oued. Crocodiles were reported to occur [3] and during this study individuals were observed in November 2003 and 2008, and April 2009. Given the proximity to guelta Matmâta and the almost permanent water flow between these two localities at the time of visits, quantifications are given ensemble (see Matmâta, locality 16).

18. Tartêga (guelta) - present

Crocodiles were reported in the permanent guelta since the 1930s [2,3,5,9]. The number of crocodiles observed have ranged throughout time, but the highest were eight crocodiles in 2000 and seven to eight in 2002 [2]. The locality was visited only by daylight in three occasions: 1) in November 2003, three to four adults were observed; 2) in November 2008, eight crocodiles were observed, including three adults and five sub-adults; and 3) in April 2009, five adult crocodiles were observed. This is one of the largest gueltas of Mauritania (about 3 ha; Figure 4A).

19. Tartêga, upstream (oued) - present

Just above guelta Tartêga, a series of small lagoons occur in the river bed. The area was visited in November 2008 only during daylight. No crocodiles were observed but faeces were found. Gueltas Bajai and M’cherba (localities 20 and 21, respectively) are within 3 km distance, following the Krâa Naga oued.

20. Bajai (oued) - present

The oued (0.05 ha) is located just 4 km above guelta Tartêga and crocodiles were known in the area [3]. In November 2008, five crocodiles were observed. Guelta M'cherba is relatively close (less than 2 km).

21. M'cherba (guelta) - present

Crocodiles were reported in the permanent guelta since 1999, when an adult (1.7 m) was found killed [2]. Two adults (1.0 and 2.0 m) and one juvenile were reported in 2000, five crocodiles (adult, sub-adult and three juveniles) in 2001, and one adult and two sub-adults in 2002 [2]. The locality was visited in two occasions: 1) in November 2008, no crocodiles were observed during day and night sampling; 2) in April 2009, one adult crocodile was observed by daylight.

22. Emreimida (guelta) - present

The permanent guelta is located in central oued Krâa Naga. In April 2009, two adults were observed swimming during daylight. The nearest locality, guelta Kabda (locality 23), is at 1 km which suggests that dispersal could occur. The guelta is overused by drinking cattle.

23. Kabda (guelta) - present

Crocodiles have been reported at the permanent guelta (0.65 ha) located in central oued Krâa Naga [3]. The area was visited in April 2009 and four crocodiles were observed by daylight. A dead adult (head: 0.27 m) was found 800 m south-east of the guelta. The animal was buried in the sandy river bed, in front of Erkenate village, and was excavated according to instructions of locals. Bone remains were recovered but the cause of death was undetermined.

24. Tin Waadine (guelta) - present

Crocodiles have been reported at the seasonal guelta (0.001 ha) since the 1930s [3,7,10], although their local extinction was suggested in 2000 [2]. The locality was visited in April 2009, but the guelta was already dry and no crocodiles were observed. However, faeces were found and several tracks heading inside rock caves were found. Crocodiles are present at the guelta but future sampling is needed to determine the number of crocodiles present.

25. Ch'Bayer (guelta) - present

The permanent guelta is located in the Krâa Naga oued. In 2000, the presence of one adult crocodile was reported and another adult (1.75 m) was found dead, about 200 m from the guelta, killed by locals [2]. In April 2009, three adults were observed during night sampling. An adult (about 1.5 m) was found dead, apparently for natural reasons, at about 900 m south-east of the guelta, in the dried river bed. Guelta Rh’ Zembou (locality 26) is at 2.5 km from Ch’Bayer which suggests that the crocodile was probably dispersing between localities.

26. Rh' Zembou (guelta) - present

The permanent guelta is located in central Krâa Naga oued and crocodiles were reported since the 1990s [1,2]. In 1993, one individual was observed, and in 2000, another adult was observed and three individuals inhabit the guelta according to locals [2]. The locality was visited in April 2009 and no crocodiles were observed inside the guelta. However, one adult (1.85 m) was found dead about 300 m east of the guelta and another dead adult (1.5 m) was observed about 2.7 km south, both killed by locals probably during dispersal events. Future sampling is needed to determine the number of crocodiles currently inhabiting the guelta.

27. Amzouzef (guelta) - present

The permanent guelta is located in the upper Krâa Naga oued and crocodiles have been reported [1,2]. In 2000, one adult and footprints were observed, and two crocodiles were present according to locals [2]. In April 2009, five crocodiles were observed during night sampling, three adults and two sub-adults.

28. Gueye (guelta) - extinct

Inquiries to locals reported the presence of crocodiles in the guelta until the 1960s [2]. After this period, crocodiles were never observed and the locality was not visited during this study. Nevertheless, the presence of crocodiles in guelta Amzouzef (locality 27), at about 8.5 km, opens the possibility of future colonisation along the Krâa Naga oued.

29. El Khedia (guelta) - present

The permanent guelta (0.15 ha) is located in the uppermost oued Krâa Naga (Figure 4B). Several crocodiles used to occur in the guelta until the 1970s, but after the droughts only one crocodile survived [review by 5]. The adult individual has been regularly observed since the 1990s [2,3]. The locality was visited in December 2007, October 2008 and April 2009, and in all occasions only one adult was observed (Figure 6C). Locals also confirm that only one crocodile is present. The nearest locality, guelta Amzouzef (locality 27), is at 37 km which suggests complete isolation of El Khedia and the future extinction of crocodiles at this locality. The guelta is heavily used by drinking cattle.

**B) Tâkhca basin**

Small endorreic basin located in north-western Tagant.

30. Sellenbou (guelta) - present

Crocodiles were reported in the guelta located in western Tagant [3]. The locality was not visited during this study. Although the guelta is relatively close to the Tâmoûrt En Na’aj basin (13.5 km; locality 8), water actually flows to the arid Moudjéria plain which results in probable isolation of the guelta.

**C) Gorgol el Abiod basin**

The main course of the Gorgol el Abiod runs about 250 km from the southern Tagant before joining the Senegal river.

31. Garaouel (guelta) - present

The presence of crocodiles was reported in the permanent guelta located in south-western Tagant in the 1930s [7]. The locality was visited on three occasions: 1) in November 2003, one adult was observed; 2) in November 2008, five adults and eleven sub-adults were observed (Figure 6D); 3) in May 2009, no individuals were observed. The guelta is composed by a series of interconnected lagoons (Figure 4C), the lower ones with an intense use by man and drinking cattle, but the upper ones being relatively undisturbed and supporting most individuals.

32 and 33. Jreif (tâmoûrt) - present

In 2007, crocodiles were reported in Toueijikjit (located along the tâmoûrt) [3], and in November 2008, faeces were found in a locality about 4 km to the south-west. The temporary tâmoûrt receives water from gueltas Garaouel and E-n-Guinâr (localities 31 and 34, respectively) and could be used by dispersing crocodiles between gueltas.

34. E-n-Guinâr (guelta) - present

The seasonal guelta is located in south-western Tagant and was visited on three occasions: 1) in December 2007, there were at least four individuals underwater that were not possible to observe directly (just air bubbles from breathing were detected). Faeces and footprints were found; 2) in November 2008, two adults were observed at night; 3) in April 2009, the water level was very low and an adult (1.8 m) crocodile was found dead at about 300 m from the guelta. No active crocodiles were observed.

35. Djouk (tâmoûrt) - possible

Locals reported in November 2003 the presence of an adult crocodile in the temporary tâmoûrt, but the individual or its signs were not observed. Also, inquiries at El Ghâira village reported that a crocodile was road-killed in the village in August 2008. The floodplain receives water from southern Tagant and northern Assaba and could assure a movement corridor between localities El Ghâira, Aouînet Nanâga, and Laout (localities 36, 37 and 38, respectively). The presence of dispersing crocodiles is probable but needs confirmation.

36. El Ghâira (source) - present

In April 2009, one adult crocodile was observed but according to locals, more than 20 crocodiles are present during the rainy season. The source is permanent and it is located in northern Assaba. Inhabitants of El Ghâira village (1.5 km from source) used the larger pools for swimming and laundry, resulting in disturbance and high levels of phosphate in water.

37. Aouînet Nanâga (source) - present

The source is located in northern Assaba, south-east of El Ghâira village. In April 2009, water availability at the source was reduced (about 200 x 10 m) and two individuals, one juvenile and one sub-adult, were found at night hiding below rock boulders (Figure S3). According to inquiries, five to six adults occur in the area during the rainy season.

38. Laout (guelta) - possible

Crocodiles were reported in the 1990s [1] at the permanent guelta located in southern Tagant, but in 2001 they were not observed [2]. The locality was visited in April 2009 and crocodiles or their signs were also not observed. Apparently populations might be extinct but see comments for Ayoun el Khechba (locality 39).

39. Ayoun el Khechba (oued) - possible

In April 2009, locals mentioned that crocodiles use the oued to move from Djouk plain up to guelta Laout (locality 38). Crocodiles or their signs were not observed during daylight sampling. Apparently, the permanent oued might be used as a corridor between the upper guelta and the lowland tâmoûrt, but the hypothesis needs confirmation.

40. Thor (guelta) - possible

The permanent guelta is located approximately 50 km to the south-west of the nearest locality with crocodiles (El Ghâira, locality 36). Locals reported in May 2009 the presence of crocodiles, but individuals or their signs were not observed. The size and configuration of the guelta suggests that presence is possible, but sampling during the rainy season is needed to confirm presence.

**D) Koûrourai basin**

The endorreic Koûrourai receives water from north-eastern Assaba and southern Tagant.

41. Oumm Icheglâne (source) - present

In April 2009, three or four juveniles were observed during night sampling. The water level was very low with only one small lagoon left (about 0.04 ha). Adults could find shelter between boulders on the base of the rocky slope (Figure S3). The source is located in central Assaba and is completely isolated from other populations.

42. Guelaga (lake) - extinct

The permanent lake is located in southern Tagant. Locals reported crocodiles until 1990s but they were extinct by hunters coming from Mali [2]. The lake was visited in April 2009 and crocodiles or their signs were not observed.

**E) Gorgol el Akhdar basin**

The main course of the Gorgol el Akhdar runs about 220 km from the western Assaba before joining the Senegal river.

43. Bâfa (oued) - present

The seasonal oued runs from western Assaba. In November 2008, a total of 11 juveniles and sub-adults were observed during night sampling. No adults were observed probably because water levels were already very low.

44. Guelleït (tâmoûrt) - possible

The tâmoûrt is located on the western foothills of the Assaba. Locals reported in November 2008 the presence of crocodiles. Although individuals were not observed, presence is highly possible given the size and preservation status of the seasonal tâmoûrt.

45. Foum Goussas (oued) - present

The seasonal oued also runs from western Assaba (Figure 5A). In November 2008, only one juvenile was observed during day sampling and one shed skin was found. No adults were observed also because the oued was almost dry. Locals mentioned also the presence of crocodiles in the nearby Le Bheyr lake, but presence was not confirmed.

46. Galoula (guelta) - possible

Crocodiles were reported in the 1950s [11] and 1970s [review by 5], when an adult (2.32 m) was collected and deposited in the Berlin Museum. The locality was not visited during this study and the current presence needs confirmation.

47. Guidemballa (guelta) - present

In November 2008, two adults were observed during daylight. There are several lagoons along the oued that flows from the main guelta, with the lowest ones having a high use by man but the upper and largest lagoon is relatively undisturbed. One crocodile was observed preying successfully over a cat-fish (*Clarias* spp.).

48. M'bout (tâmoûrt) - possible

Crocodiles were reported in the surroundings of M’bout, about 70 km south-west of Assaba, in the 1930s [7]. The locality was not visited during this study. Given that a dam was built north of M’bout, crocodile presences remains possible but needs confirmation.

**F) Oued Garfa basin**

The main course of the Oued Garfa basin runs about 140 km from south-western Assaba before joining the Senegal river.

49. Soungount (guelta) - possible

At least 30 crocodiles were reported in the guelta in 1955 [12]. The locality was not visited during this study and surveys are needed to confirm the current presence.

50. Goumbel (guelta) - present

The permanent guelta is located in extreme south-western Assaba. In November 2008, five crocodiles were observed during night sampling and the lower jaw of a sub-adult was found. The 9 km that separate the guelta from tâmoûrt Guenétir (locality 51) cross a vast seasonal floodplain which opens the possibility of dispersal between populations.

51. Guenétir 1 (tâmoûrt) - possible

The tâmoûrt is a vast seasonal floodplain (about 440 ha) located on the south-western foothills of Assaba. The locality was visited in two occasions, December 2007 and November 2008. Although crocodiles were not observed in both occasions, locals reported their presence. The tâmoûrt was nearly dry in both visits offering few opportunities to find active crocodiles.

52. Guenétir 2 (source) - present

A small source contiguous to the tâmoûrt, on the Assaba rocky slope, was visited on December 2007 and November 2008. Crocodiles were not observed, but faeces were found in December 2007. The water level was very low probably hampering activity. Locals reported that crocodiles were present. The same potential preys detected in the tâmoûrt (locality 51) were found in the source.

**G) Karakoro basin**

The main course of the Karakoro runs about 400 km from the eastern Assaba and western Affolé before joining the Senegal river.

53. Kankossa (lake) - possible

Crocodiles were reported in the 1950s [11]. In December 2007 locals reported in the presence of crocodiles but they were not observed. Given the relative large size of the lake (about 120 ha), it is possible that crocodiles are present. There is a large pressure over water, with several hundreds of cows, dromedaries and goats drinking daily from the lake.

54. Legleyta (guelta) - present

In April 2009, two adult crocodiles (about 2.0 m) were found hidden inside a rock cave with about 8 m long (Figure 6E). The guelta was dry and animals found shelter between the rock boulders that surround the pool. Several tracks and footprints were observed above the main guelta. Locals reported that crocodiles are abundant during the rainy season.

55. Mendjoura (guelta) - extinct

In April 2009, locals reported crocodiles until the 1970s, when severe droughts dried the guelta for several years consecutively. No crocodiles or their signs were observed at the lowest part of the guelta. It were observed fishes (*Barbus spp*.), amphibians (*Hoplobatrachus occipitalis*) and *V. niloticus*, suggesting that smaller-sized vertebrates were able to survive the severe droughts.

56. Boû blei'îne (lake) - present

In April 2009, three adult crocodiles were observed during daylight in a small island of the lake. Given the large area (1,600 ha) of the lake (Figure 5B) and its permanent character, it is probable that more individuals are present. The nearest locality with crocodiles, Tâmoûrt Taghtâfet (locality 57), is at about 40 km distance.

57. Taghtâfet (tâmoûrt) - present

In October 2008, an adult was observed basking in the water margin and locals reported that they were common in the seasonal tâmoûrt (Figure 5C). One sub-adult crocodile (about 1.3 m) was found dead by undetermined causes and a second adult (1.2 m) was found killed by locals at north-west of Taghtâfet (Figure 6F). The area is a series of parallel channels, after the ending of the tâmoûrt, and the crocodile remains were found at 350 m from the shoreline. It can be hypothesised that the dispersing individual was returning to the main lagoon, probably making a shortcut through a sandy area (1.2 km instead of 3.4 km if following the main course). This observation suggests that it is possible the occurrence of dispersal with tâmoûrts Jaraaziza and Tâmchekket (localities 58 and 59, respectively), located at about 8 km south and connected by a seasonal oued.

58. Jaraaziza (tâmoûrt) - present

At the extreme north-west of tâmoûrt Tâmchekket (locality 59), the main tâmoûrt closes and forms as series of relatively narrow channels. In October 2008, no crocodiles were observed during day or night sampling but many faeces and footprints were found. At the time of visit, the tâmoûrt was almost dry and apparently crocodiles were no longer active. Locals reported that during the rainy season, crocodiles are abundant.

59. Tâmchekket (tâmoûrt) - present

Several authors reported the presence of crocodiles in the seasonal tâmoûrt (400 ha) [2,10, 13,14,15]. In October 2008, about 15 to 20 crocodiles were observed during night sampling, including adults, sub-adults and juveniles. Locals reported that crocodiles occasionally capture goats but not cows or dromedaries, and when water dries, crocodiles find shelter in burrows made in the dry muddy banks. The main lagoon closer to Tâmchekket village is overused by drinking cattle, but the remaining areas are relatively preserved.

60. Megta es Sfeira (dam) - present

Crocodiles were reported in the 1950s [11]. The permanent dam was visited in two occasions: 1) in December 2007, locals reported that crocodiles were present but they were not observed; 2) in November 2008, two individuals (adult and sub-adult) were observed swimming at daylight.

61. Gâdoum (tâmoûrt) - present

In November 2008, one large adult (estimated to have over 2.5 m) was observed at daylight in a small island of the seasonal tâmoûrt, eating an adult *V. niloticus*. The nearest population, Megta es Sfeira dam (locality 60), is at about 4 km distance. When water becomes scarce, the locality is under enormous pressure from man and drinking cattle.

62. Bougâri (tâmoûrt) - present

Crocodiles were reported in the seasonal tâmoûrt (Figure 5D) [14,16]. In November 2008, one adult was observed at daylight and another adult (1.3 m) was found dead, killed by locals, at about 200 m from the shoreline. Locals reported that crocodiles find shelter in large excavated burrows when the tâmoûrt dries (Figure S2).

63. Metraoucha (guelta) - present

Crocodiles were recently reported in the permanent guelta [14,16]. The locality was visited in two occasions: 1) in December 2007, about 15 to 20 crocodiles were observed at daylight, including adults and sub-adults (Figure 6G); 2) in October 2008, more than 20 crocodiles were observed at daylight. This is one of the gueltas with the largest number of individuals recorded. The guelta is formed by two well preserved lagoons followed by an oued relatively disturbed by drinking cattle. Tâmoûrt Bougâri and guelta Oumm el Mhâr (localities 62 and 64, respectively) are relatively close, 6 km and 7 km, respectively.

64. Oumm el Mhâr (guelta) - present

The permanent guelta was visited in two occasions: 1) in December 2007, only one adult emerging the head for a brief moment was observed at daylight; 2) in October 2008, two adults were observed during daylight and 18 crocodiles, including adults, sub-adults and juveniles, were observed during night sampling. Eight individuals were observed in the guelta and 10 in the oued that flows from the guelta. The oued was almost dried and most crocodiles were observed hidden under rock boulders. The small sized guelta (less than 0.025 ha) is overexploited by drinking cattle (Figure 4D).

**H) Mefga basin**

The Mefga basin is an endorreic system located in central Affolé.

65. El Mefga (guelta) - present

The permanent guelta was sampled in October 2008 and 10 crocodiles were observed, two adults and eight juveniles. The guelta was small (less than 0.08 ha) and crocodiles found shelter during the day under a huge boulder on the rocky slope. There is an overuse of water, with dozens of cows, dromedaries and goats invading the lagoon throughout the day. One crocodile was found dead 2.5 km east of the guelta. The animal was buried in the sandy river bed and was excavated according to instructions of locals. Bone remains were recovered and apparently another dead crocodile was present in the area but was not found. Tâmoûrt Kour (locality 66) is at about 2 km to the north of the guelta which suggests the possibility of dispersal between localities.

66. Kour (tâmoûrt) - present

Crocodiles were recently reported in the permanent tâmoûrt [15]. The locality was visited in October 2008 and four adult crocodiles were observed swimming in the lagoon (250 ha).

**I) Tâyâret el Msîlé basin**

The Tâyâret el Msîlé basin is a vast endorreic basin located in south-eastern Mauritania.

67. Chegg el Mâleh (source) - present

The permanent source is located in a vast and open floodplain (550 ha) on the southern face of the Affolé. A water trough has been built over the source to allow cattle to drink during the dry season. Since the tâmoûrt is seasonal, crocodiles find shelter inside the trough. Although the structure is small (about 20 x 2 m, Figure S3), in December 2007, six juveniles were observed at sunset, and in October 2008, one adult (about 2.0 m) and two sub-adults were observed during night sampling. During the day, crocodiles rest submerged while hundreds of cows and dromedaries come to drink, but at night bask over rocks boulders inside the trough. Prey availability appears to be highly limited in time. In November, there were hundreds of active *H. occipitalis* inside and outside of the trough (Figure 6H), but many more were already hiding in nearby rock outcrops. In December, no amphibians were detected and the area was already dry. No other potential prey was detected in November. Thus, the feeding period of crocodiles should be strictly concentrated to the active period of amphibians (probably from August to November).

68. Lemsille Gharghar (tâmoûrt) - present

Crocodiles were reported in the seasonal tâmoûrt (2,000 ha) located at about 30 km south of the Affolé [15]. The locality was not visited during this study.

69. Guelb Samba (dam) - present

The permanent dam is located in eastern Affolé. In October 2008, two crocodiles, one adult and one sub-adult, were observed during both day and night sampling. Locals reported that in the past, it was usual to observe six individuals. Reasons for apparent decline are unknown.

70. Rachida (tâmoûrt) - present

Crocodiles were reported in the seasonal tâmoûrt (180 ha) located at about 30 km east of the Affolé [15]. The locality was not visited during this study.

71. Chelkha (guelta) - present

Crocodiles were reported in the guelta located in southern Affolé [15]. The locality was not visited during this study.

72. Goungel (tâmoûrt) - present

Several authors reported crocodile presence in the seasonal tâmoûrt [10,14,15]. In October 2008, one adult was observed at night. Given the large size of the tâmoûrt (530 ha) it is possible that many more crocodiles are present. Locals reported that during the rainy season, crocodiles leave the large lake at night and prey on domestic animals in the nearby village.

73. Oum Lelli (tâmoûrt) - present

Crocodiles were reported in the seasonal tâmoûrt (350 ha) located in the extreme south-eastern Affolé [15]. The locality was not visited during this study.

74. Gaât Sawana (tâmoûrt) - present

Crocodiles were reported in the seasonal tâmoûrt (1,100 ha) located in extreme south-eastern Affolé [15]. The locality was not visited during this study.

75. Oum Azvavail (tâmoûrt) - present

Crocodiles were reported in the seasonal tâmoûrt (1,500 ha) [15]. The locality was not visited during this study.

76. Gaât Touil (tâmoûrt) - present

Crocodiles were reported in the seasonal tâmoûrt (1,200 ha) located at about 40 km from the Malian border [15]. The locality was not visited during this study.

77. Ould Agueila (oued) - present

Crocodiles were reported in the seasonal oued (4,000 ha) located at about 13 km from the Malian border [15]. The locality was not visited during this study.

**J) Nioût basin**

The Nioût basin is an endorreic system located in extreme south-eastern Mauritania.

78. Dendaré (lake) - possible

Crocodiles were reported in the 1930s [4,7]. The locality was not visited during this study and current presence needs confirmation.

**References**

1. Behra O (1994) Crocodiles on the desert's doorstep. Crocodile Specialist Group Newsletter 13: 4-5.

2. Lluch P, Robin S, Lescure J (2004) Le Crocodile du Nil, *Crocodylus niloticus* Laurenti, 1768 dans le Tagant (Mauritanie). Bulletin de la Société Herpétologique de France111-112: 5-23.

3. Tellería JL, Ghaillani HEM, Fernández-Palacios JM, Bartolomé J, Montiano E (2008) Crocodiles *Crocodylus niloticus* as a focal species for conserving water resources in Mauritanian Sahara. Oryx 42: 292-295.

4. Padial JM (2006) Commented distributional list of the reptiles of Mauritania (West Africa). Graellsia 62: 159-178.

5. de Smet K (1999) Status of the Nile crocodile in the Sahara desert. Hydrobiologia 391: 81-86.

6. Staudinger P (1928) Krokodile in der inner-Sahara und Mauritanien. Sitz. Ber. Ges. Naturf. Fr. 4: 141-142.

7. Joleaud L (1933) Étude de géographie zoologique sur la Berbérie. Les Reptiles - Les Crocodiliens. Bulletin de la Société Zoologique de France 58: 397-403.

8. Sahara Conservation Fund (2007) Saharan crocs past and present. Sandscript 2: 4.

9. Monod Th (1937) Méharées. Explorations au vrai Sahara. Ed. Jesers, Paris. 303 p.

10. Shine T, Böhme W, Nickel H, Thies DF, Wilms T (2001) Rediscovery of relict populations of the Nile crocodile *Crocodylus niloticus* in south-eastern Mauritania, with observations on their natural history. Oryx 35: 260-262.

11. Munier P (1952) L'Assaba. Essai Monographique. Études Mauritaniennes 3: 1-71.

12. Roberty G (1958) Végétation de la guelta de Soungout (Mauritanie méridionale), en mars 1955. Bulletin de l'Institut Fondamental d'Afrique Noire 20: 869-875.

13. National Research Council (1981) Environmental Degradation in Mauritania. Board on Science and Technology for International Development. Commission on International Relations. National Academy Press, Washington.

14. Cooper A, Shine T, McCann T, Tidane, TA (2006) An ecological basis for sustainable land use of Eastern Mauritanian wetlands. Journal of Arid Environments 67: 116-141.

15. Kirsch-Jung KP, Khtour DO (2007) Conservation et utilisation des zones humides dans le Hodh El Gharbi mauritanien. République Islamique de Mauritanie, Secrétariat d'Etat auprès du Premier Ministre chargé de l'Environnement and Coopération Technique Allemande (GTZ). 109 p. Available: http://www2.gtz.de/dokumente/bib/07-0608.pdf

16. Nickel H (2003) Ökologische untersuchungen zur wirbeltierfauna im südöstlichen Mauretanien. Zwei fallstudien unter berücksichtigung der Krokodile. GTZ, Eschborn. 89 p. Available at: http://www2.gtz.de/dokumente/bib/04-5502.pdf.
