# Supplementary material for: Crocodiles in the Sahara Desert: An Update of Distribution, Habitats and Population Status for Conservation Planning in Mauritania
Source: PLoS One. 2011 Feb 25;6(2):e14734. doi: 10.1371/journal.pone.0014734 (PMC3045445; doi:10.1371/journal.pone.0014734)
Supplement: Table S1 — Distribution, status and date of last observation of Nile crocodile populations in the Sahara excluding extant localities for Mauritania. (0.10 MB DOC) [file pone.0014734.s002.doc]

**Table 1.** Distribution, status and date of last observation of Nile crocodile populations in the Sahara excluding extant localities for Mauritania.

|  |  |  |  |  |  |
| --- | --- | --- | --- | --- | --- |
| **Country and locality** | **Latitude** | **Longitude** | **Date** | **Status** | **Author(s)** |
| **Algeria** |  |  |  |  |  |
| **I-n-Houter** | 23.12833 | 5.35833 | 1950s | EX | [18,25] |
| **Oued Ihérir-Imihrou** | 25.76793 | 8.65783 | 1924 (1946?) | EX | [18,23,25,55] |
| **Oued Tedjoujelt** | 26.54233 | 7.83907 | 1858 | EX | [18,23] |
| **I-n-Tawinast** | 26.01000 | 4.62333 | 1984 | EX | [25] |
| **Algiers, close to** | 36.51352 | 3.06570 | 2000 BP | HOL | [18] |
| **Meniet** | 24.99000 | 4.33000 | 5400 BP | HOL | [18] |
| **Oua n’Rechla** | 20.43000 | 5.20000 | 9000 BP | HOL | [18] |
| **I-n-Guezzâm** | 19.44500 | 5.81200 | - | HOL | [46] |
| **Tihodaine** | 25.30000 | 6.90000 | - | HOL | [46] |
| **Tahag** | 23.29000 | 5.55000 | - | HOL | [46] |
| **Chad** |  |  |  |  |  |
| **Guelta Archei** | 16.89887 | 21.77653 | 2000s | PR | [18,22,56,60,64] |
| **Guelta Tottous** | 19.44000 | 17.52000 | 1920s | UN | [18,56] |
| **Gueltas of Tibesti** | 21.00000 | 17.00000 | 1800s | UN | [18] |
| **Oudougeï** | 19.36000 | 17.54000 | 1930 | UN | [18,56] |
| **Ounianga Kebir** | 19.05000 | 20.50000 | 4000 BP | HOL | [45] |
| **Egypt** |  |  |  |  |  |
| **Lake Nasser** | 23.56000 | 32.86000 | 2006 | PR | [52] |
| **Nile Delta** | 31.21966 | 31.12313 | 1753 | EX | [52] |
| **Bir Tarfawi** | 22.92700 | 28.87800 | - | HOL | [50] |
| **Fayoum depression** | 29.33000 | 31.20000 | 3000–2600 BP | HOL | [52] |
| **Wadi el-Barramiya** | 25.03333 | 33.65833 | 4000 BP | HOL | [51] |
| **Libya** |  |  |  |  |  |
| **Djebel Akhdar** | 32.19618 | 21.41950 | 2000 BP | HOL | [18] |
| **In Habiter** | 26.49082 | 12.69354 | unknown | HOL | [32] |
| **Wadi Matendous** | 25.76267 | 12.16647 | unknown | HOL | [authors, unpub. data] |
| **Mali** |  |  |  |  |  |
| **Taoudeni** | 22.62000 | -4.05000 | 5400–4000 BP | HOL | [18,32] |
| **Tessalit** | 20.29679 | 0.96720 | 2000 BP | HOL | [32] |
| **Erg Ine Sakane/Takabart** | 20.55955 | -0.96619 | 5750 BP | HOL | [48] |
| **Hassi el Abiod** | 19.19248 | -3.82369 | 5750-5000 BP | HOL | [48,49] |
| **Mauritania** |  |  |  |  |  |
| **Chinguetti** | 20.47522 | -12.33861 | unknown | HOL | [43] |
| **Oum Aghouâba** | 21.10000 | -12.00000 | 2000 BP | HOL | [9,18] |
| **Bou Khzama** | 16.73667 | -7.27100 | 4000–2300 BP | HOL | [54] |
| **Ouarâne** | 20.86000 | -9.66000 | unknown | HOL | [44] |
| **Dhar Tîchît** | 18.42000 | -9.52000 | 4500–3500 BP | HOL | [54] |
| **Morocco** |  |  |  |  |  |
| **Guelta Taffagount** | 29.19508 | -8.25073 | 1950s | EX | [18,57,58] |
| **Guelta Tanzida** | 28.81290 | -8.90797 | 1950s | EX | [58] |
| **Tiglite, SW of** | 28.40188 | -10.40342 | 1930s | EX | [59] |
| **Tizgui Remz** | 28.41228 | -9.21762 | 1950s | EX | [18,57,58] |
| **Niger** |  |  |  |  |  |
| **Adrar Bous** | 20.36000 | 9.02000 | 5500 BP | HOL | [18,47] |
| **Gobero** | 19.91580 | 9.49701 | 5200 BP | HOL | [53] |
| **Sudan** |  |  |  |  |  |
| **Wadi Howar** | 15.93369 | 24.34542 | 5200 BP | HOL | [18] |
| **Tunisia** |  |  |  |  |  |
| **Chott el Djerid** | 33.70000 | 8.30000 | 1921 | EX | [18] |
| **Western Sahara** |  |  |  |  |  |
| **Seguiet el Hamra** | 26.80000 | -12.20000 | 2000 BP | HOL | [18,32,42] |

Coordinates (WGS84 projection) are in decimal degrees. Status: PR – present; UN – unknown; EX – extinct; HOL – present in Holocene
