# Supplementary material for: Crocodiles in the Sahara Desert: An Update of Distribution, Habitats and Population Status for Conservation Planning in Mauritania
Source: PLoS One. 2011 Feb 25;6(2):e14734. doi: 10.1371/journal.pone.0014734 (PMC3045445; doi:10.1371/journal.pone.0014734)
Supplement: Table S2 — Distribution, status and date of last observation of Nile crocodile populations in Mauritania. (0.16 MB DOC) [file pone.0014734.s003.doc]

**Table 2.** Distribution, status and date of last observation of Nile crocodile populations in Mauritania.

|  |  |  |  |  |  |  |  |  |
| --- | --- | --- | --- | --- | --- | --- | --- | --- |
| **Basin/Local** | **Type** | **Latitude** | **Longitude** | **Date** | **Water** | **Status** | **N** | **Author(s)** |
| **A) Gabbou** |  |  |  |  |  |  |  |  |
| **1. Ederoum** | D | 18.32678 | -11.55292 | 2008 | P | PR | 5 | [26,29,*] |
| **2. Motoboul** | G | 18.48324 | -11.74083 | 2007 | S | PR | - | [31] |
| **3. Taorta** | O | 18.20358 | -11.73466 | 2009 | JAN | PR | - | [31,*] |
| **4. Kaimel** | G | 18.15351 | -12.01259 | 2007 | P | PR | - | [31,*] |
| **5. Daal** | G | 18.38643 | -12.16497 | 2007 | S | PR | - | [31,*] |
| **6. Gabbou** | L | 18.27788 | -12.36465 | 2007 | P | PR | - | [31,*] |
| **7. Dekheïlet el ‘Aleïb** | L | 18.07665 | -12.31629 | 2007 | P | PR | - | [31] |
| **8. En Na'aj** | T | 17.94294 | -12.25698 | 2009 | P | PR | - | [31,*] |
| **9. Fanar** | G | 18.01585 | -12.17497 | 2000s | S | NC | - | [61,*] |
| **10. Marshra** | L | 17.88457 | -12.18455 | 2007 | P | NC | - | [31,*] |
| **11. Suklan** | G | 17.84979 | -12.18643 | 2008 | JAN | PR | - | [31,*] |
| **12. Tkhsutin** | G | 17.82126 | -12.18576 | 2008 | P | PR | - | [31,*] |
| **13. Bourâgga** | L | 17.76879 | -12.22982 | 2008 | P | NC | - | [31,*] |
| **14. El Housseînîya** | S | 17.73796 | -12.24525 | 2009 | P | PR | - | [31,*] |
| **15. Dâber** | G | 17.57129 | -12.17857 | 2007 | P | NC | - | [31,*] |
| **16. Matmâta** | G | 17.88730 | -12.11084 | 2009 | P | PR | 10 | [18,20,26,28,29,31,32,*] |
| **17. Jabara** | G | 17.88730 | -12.10098 | 2007 | S | PR | - | [31,*] |
| **18. Tartêga** | G | 17.88108 | -12.09400 | 2009 | P | PR | 8 | [18,29,31,*] |
| **19. Tartêga, upstream** | O | 17.87557 | -12.09165 | 2008 | P | PR | - | [*] |
| **20. Bajai** | O | 17.84634 | -12.07824 | 2008 | P | PR | 5 | [31,*] |
| **21. M'cherba** | G | 17.85531 | -12.06884 | 2009 | P | PR | 5 | [29,*] |
| **22. Emreimida** | G | 17.85244 | -11.97194 | 2009 | P | PR | 2 | [*] |
| **23. Kabda** | G | 17.84638 | -11.96210 | 2009 | P | PR | 4 | [31,*] |
| **24. Tin Waadine** | G | 18.05349 | -11.94289 | 2009 | APR | PR | - | [27,29,31,32,*] |
| **25. Ch'Bayer** | G | 17.76208 | -11.88283 | 2009 | P | PR | 3 | [29,*] |
| **26. Rh' Zembou** | G | 17.74163 | -11.87386 | 2009 | P | PR | 1 | [26,29,*] |
| **27. Amzouzef** | G | 17.70607 | -11.82615 | 2009 | P | PR | 5 | [26,29,*] |
| **28. Gueye** | G | 17.67004 | -11.76374 | 1960s | S | EX | - | [29] |
| **29. El Khedia** | G | 17.83485 | -11.55783 | 2009 | P | PR | 1 | [18,29,31,*] |
| **B) Tâkhca** |  |  |  |  |  |  |  |  |
| **30. Sellenbou** | G | 17.81617 | -12.27857 | 2007 | U | PR | - | [31] |
| **C) Gorgol el Abiod** |  |  |  |  |  |  |  |  |
| **31. Garaouel** | G | 17.45167 | -12.39485 | 2009 | P | PR | 11 | [32,*] |
| **32. Jreif (=Toueijikjit)** | T | 17.42817 | -12.41987 | 2007 | JAN | PR | - | [31] |
| **33. Jreif** | T | 17.40211 | -12.38884 | 2008 | JAN | PR | - | [*] |
| **34. E-n-Guinâr** | G | 17.40143 | -12.36415 | 2009 | MAY | PR | 4 | [*] |
| **35. Djouk** | T | 17.23405 | -12.26003 | 2003 | APR | PO | - | [*] |
| **36. El Ghâira** | S | 17.18834 | -12.24810 | 2009 | P | PR | 1 | [*] |
| **37. Aouînet Nanâga** | S | 17.15248 | -12.19912 | 2009 | JAN | PR | 2 (6) | [*] |
| **38. Laout** | G | 17.24083 | -12.10167 | 1990s | P | NC | - | [26,*] |
| **39. Ayoun el Khechba** | O | 17.23279 | -12.10001 | 2009 | P | PO | - | [*] |
| **40. Thor** | G | 17.06988 | -12.68863 | 2009 | P | PO | - | [*] |
| **D) Koûrourai** |  |  |  |  |  |  |  |  |
| **41. Oumm Icheglâne** | S | 17.07030 | -12.20785 | 2009 | P | PR | 3-4 | [*] |
| **42. Guelaga** | L | 17.25267 | -11.97012 | 2009 | P | EX | - | [29,*] |
| **E) Gorgol el Akhdar** |  |  |  |  |  |  |  |  |
| **43. Bâfa** | O | 16.88873 | -12.18487 | 2008 | JAN | PR | 11 | [*] |
| **44. Guelleït** | T | 16.73176 | -12.16877 | 2008 | DEC | PO | - | [*] |
| **45. Foum Goussas** | O | 16.54746 | -12.00959 | 2008 | DEC | PR | 1 | [*] |
| **46. Galoula** | G | 16.33941 | -11.97777 | 1970s | U | PO | - | [18,33] |
| **47. Guidemballa** | G | 16.18484 | -12.01206 | 2008 | P | PR | 2 | [*] |
| **48. M'bout** | D | 16.06207 | -12.60034 | 1930s | U | PO | - | [32] |
| **F) Oued Garfa** |  |  |  |  |  |  |  |  |
| **49. Soungount** | G | 15.9778 | -12.0088 | 1955 | U | PO | 30 | [29,34] |
| **50. Goumbel** | G | 15.95708 | -12.00986 | 2008 | P | PR | 5 | [*] |
| **51. Guenétir 1** | T | 15.87822 | -12.03923 | 2007 | DEC | PO | - | [*] |
| **52. Guenétir 2** | S | 15.88325 | -12.03637 | 2007 | P | PR | - | [*] |
| **G) Karakoro** |  |  |  |  |  |  |  |  |
| **53. Kankossa** | L | 15.93160 | -11.54413 | 2007 | P | PO | - | [33,*] |
| **54. Legleyta** | G | 16.75648 | -11.99723 | 2009 | MAR | PR | 2 | [*] |
| **55. Mendjoura** | G | 17.45396 | -11.50110 | 2009 | S | EX | - | [*] |
| **56. Boû blei'îne** | L | 17.14076 | -11.01114 | 2009 | P | PR | 3 | [*] |
| **57. Taghtâfet** | T | 17.32917 | -10.70857 | 2008 | DEC | PR | - | [*] |
| **58. Jaraaziza** | T | 17.26138 | -10.69015 | 2008 | NOV | PR | - | [*] |
| **59. Tâmchekket** | T | 17.24986 | -10.66761 | 2008 | DEC | PR | 15-20 | [26,29,35,39,62,*] |
| **60. Megta es Sfeira** | D | 16.64037 | -11.05618 | 2008 | P | PR | 2 | [33,*] |
| **61. Gâdoum** | T | 16.60610 | -11.05539 | 2008 | JAN | PR | 1 | [*] |
| **62. Bougâri** | T | 16.54009 | -10.80149 | 2008 | S | PR | 1 | [28,39,*] |
| **63. Metraoucha** | G | 16.53803 | -10.74155 | 2008 | P | PR | >20 | [28,39,*] |
| **64. Oumm el Mhâr** | G | 16.57915 | -10.70455 | 2008 | P | PR | 18 | [*] |
| **H) Mefga** |  |  |  |  |  |  |  |  |
| **65. El Mefga** | G | 16.68758 | -10.19136 | 2008 | P | PR | 10 | [*] |
| **66. Kour** | T | 16.70180 | -10.18359 | 2008 | P | PR | 4 | [62,*] |
| **I) Tâyâret el Msîlé** |  |  |  |  |  |  |  |  |
| **67. Chegg el Mâleh** | S | 16.51556 | -10.45291 | 2008 | JAN | PR | 6 | [*] |
| **68. Lemsille Gharghar** | T | 16.15233 | -10.35667 | 2000s | FEB | PR | - | [62] |
| **69. Guelb Samba** | D | 16.65499 | -9.70784 | 2008 | P | PR | 2 | [*] |
| **70. Rachida** | T | 16.67533 | -9.30600 | 2000s | JAN | PR | - | [62] |
| **71. Chelkha** | G | 16.42538 | -9.63071 | 2000s | P | PR | - | [62] |
| **72. Goungel** | T | 16.40315 | -9.55986 | 2008 | APR | PR | 1 | [26,39,62,*] |
| **73. Oum Lelli** | T | 16.38470 | -9.30056 | 2000s | MAY | PR | - | [62] |
| **74. Gaât Sawana** | T | 16.29671 | -9.25699 | 2000s | FEB | PR | - | [62] |
| **75. Oum Azvavail** | T | 15.85783 | -9.01683 | 2000s | MAR | PR | - | [62] |
| **76. Gaât Touil** | T | 15.74516 | -9.68080 | 2000s | FEB | PR | - | [62] |
| **77. Ould Agueila** | O | 15.50483 | -9.81683 | 2000s | MAR | PR | - | [62] |
| **J) Nioût** |  |  |  |  |  |  |  |  |
| **78. Dendaré** | L | 16.28670 | -6.68688 | 1930 | U | PO | - | [32,61] |

Type refers to the category of water locality; Date refers to the year or decade of last observation; Water refers to the yearly availability of water; Status refers to population status; N refers to the maximum number of crocodiles reported. Coordinates (WGS84 projection) are in decimal degrees. Type: D – Dam; G – Guelta; L – Lake; O – Oued; S – Source; T – Tâmoûrt. Water: P – Permanent; S – Seasonal; U – Unknown. When known, the month when the locality dries is given. Status: PR – Present; PO – Possible; NC – Previously reported but not confirmed in 2008 or 2009; EX – Extinct (see methods for details). * - this study.
